# Exceptional Heme Tolerance in *Serratia plymuthica*: Proteomic Insights into Oxidative Stress Adaptation in the *Aedes aegypti* Midgut

**DOI:** 10.3390/life15060950

**Published:** 2025-06-13

**Authors:** Sâmella da Hora Machado, Rívea Cristina Custódio Rodrigues, Maria Aparecida Aride Bertonceli, Analiz de Oliveira Gaio, Gabriela Petroceli-Mota, Ricardo de Souza Reis, Marília Amorim Berbert-Molina, Vanildo Silveira, Francisco José Alves Lemos

**Affiliations:** 1 Laboratorio de Biotecnologia, Centro de Biociências e Biotecnologia, Universidade Estadual do Norte Fluminense Darcy Ribeiro (UENF), Campos dos Goytacazes 28013-602, RJ, Brazil; horasamy@yahoo.com.br (S.d.H.M.); rivea@uenf.br (R.C.C.R.); analizgaio@yahoo.com.br (A.d.O.G.); ricardoreisreis@gmail.com (R.d.S.R.); mberbert@uenf.br (M.A.B.-M.); vanildo@uenf.br (V.S.); 2 Laboratorio de Química e Função de Proteinas e Peptídeos, Centro de Biociências e Biotecnologia, Universidade Estadual do Norte Fluminense Darcy Ribeiro (UENF), Campos dos Goytacazes 28013-602, RJ, Brazil; cidaride@hotmail.com; 3 Laboratorio de Biologia Celular e Tecidual, Centro de Biociências e Biotecnologia, Universidade Estadual do Norte Fluminense Darcy Ribeiro (UENF), Campos dos Goytacazes 28013-602, RJ, Brazil; gabrielapetroceli@gmail.com; 4 Unidade de Biologia Integrativa, Setor de Genômica e Proteômica, Universidade Estadual do Norte Fluminense Darcy Ribeiro (UENF), Campos dos Goytacazes 28013-602, RJ, Brazil

**Keywords:** *Aedes aegypti* midgut, *Serratia plymuthica*, heme stress, oxidative stress adaptation, proteomics

## Abstract

*Serratia plymuthica*, isolated from the midgut of *Aedes aegypti*, displays remarkable resilience to hemin, a toxic hemoglobin byproduct generated during blood digestion. This study explores its proteomic adaptations under oxidative stress induced by 5 mM hemin, mimicking midgut conditions. Growth assays demonstrated that *S. plymuthica* tolerated hemin concentrations ranging from 5 µM to 1 mM, reaching the stationary phase within approximately 10 h. Colonies exhibited morphological changes—darkened peripheries and translucent halos—suggesting heme accumulation and detoxification. Label-free quantitative proteomics identified 436 proteins, among which 28 were significantly upregulated—including universal stress proteins (USPs), ABC transporters, and flavodoxin—while 54 were downregulated, including superoxide dismutase and several ribosomal proteins. Upregulated proteins were associated with antioxidant defense, heme transport, and redox regulation, whereas downregulated proteins suggested metabolic reprogramming to conserve energy under stress. Functional enrichment analysis revealed significant alterations in transmembrane transport, oxidative stress response, and central metabolism. These findings suggest that *S. plymuthica* contributes to redox homeostasis in the mosquito gut by mitigating reactive oxygen species (ROS) and detoxifying excess heme, supporting its role as a beneficial symbiont. The observed stress tolerance mechanisms may influence mosquito physiology and vector competence, offering novel insights into mosquito–microbiota interactions and potential microbiota-based strategies for vector control.

## 1. Introduction

Mosquitoes of the genus *Aedes* are primary vectors of arboviruses such as dengue, Zika, and chikungunya [1]. Their ability to transmit pathogens is closely associated with their hematophagous behavior, which presents metabolic challenges, particularly during blood digestion [2,3]. One major challenge is the oxidative stress generated by heme, the prosthetic group of hemoglobin, which catalyzes the formation of reactive oxygen species (ROS) [4]. Excess heme can lead to cellular damage through lipid peroxidation and protein oxidation, necessitating specialized detoxification mechanisms in mosquitoes [5,6,7]. During blood digestion, heme concentrations in the gut lumen of *A. aegypti* can reach approximately 10 mM [8], creating a highly oxidative environment that exerts strong selective pressure on resident microbial communities.

The midgut of *A. aegypti* hosts a diverse microbiota involved in digestion, nutrient assimilation, and immune modulation [9,10]. Among these microbial inhabitants, bacteria of the genus *Serratia* are of particular interest due to their dominance in this oxidative environment [11]. Although most studies have characterized *Serratia* spp. isolated from plants, highlighting traits such as phytohormone production, nutrient solubilization, antioxidant activity, and modulation of host gene expression [12], these features may also be advantageous in the mosquito midgut. In particular, genomic analyses of plant-associated *S. plymuthica* strains have revealed the presence of robust oxidative stress-response systems, including superoxide dismutase (sodA), catalases (katA/G), and glutathione S-transferases, which neutralize ROS and mitigate heme toxicity [13]. These capabilities suggest that *S. plymuthica* could contribute to redox balance and heme homeostasis within the mosquito gut.

While mosquitoes have evolved antioxidant mechanisms, such as catalase induction [4], to mitigate heme toxicity, midgut-associated bacteria may act synergistically to maintain redox homeostasis. For instance, heme-peroxidases in mosquitoes regulate bacterial populations by modulating ROS levels and mucin crosslinking, creating micro-niches that supports microbiota proliferation [14]. Certain *Serratia* species produce antioxidant molecules, indirectly protecting associated hosts (e.g., mosquitoes) from oxidative damage while enhancing bacterial survival [15,16]. These interkingdom interactions are essential for sustaining microbial equilibrium and can influence pathogen susceptibility, as dysbiosis in the gut microbiota is known to affect Plasmodium and arbovirus infections [17].

Understanding the physiological and molecular adaptations of *S. plymuthica* to heme-induced oxidative stress is crucial for unraveling its survival strategies and potential role in mosquito–microbiota interactions. Following blood ingestion, the gut environment becomes highly oxidative, compelling bacterial symbionts to activate protective mechanisms that ensure both survival and functional stability [14]. We hypothesize that *S. plymuthica* plays an active role in mitigating oxidative stress in the mosquito gut, which may impact microbial homeostasis and vector competence.

In this study, we evaluate the growth and stress tolerance of *S. plymuthica* in response to varying concentrations of heme and iron in culture media. Using a label-free quantitative proteomics approach, we identify differentially accumulated proteins and key metabolic pathways associated with oxidative stress resistance. Particular attention is given to universal stress proteins, ABC transporters, and other redox-regulatory proteins involved in adaptation to heme toxicity. These findings provide novel insights into the ecological role of *S. plymuthica* within the mosquito gut and its potential impact on host physiology and vectorial capacity. Furthermore, they open new avenues for the development of microbiota-based strategies for vector control and biotechnological exploitation of heme-tolerant bacteria.

## 2. Material and Methods

### 2.1. Microorganism and Culture Conditions

*S. plymuthica* was isolated from the midgut of adult female *A. aegypti* mosquitoes, which were blood-fed 24 h prior to dissection to reflect the oxidative environment encountered during heme metabolism. For blood feeding experiments, mosquitoes were fed using gently immobilized Balb/c mice, following approved ethical protocols (CEUA/CBB/UENF approval no. 011/2023), in accordance with the guidelines established by the National Council for Control of Animal Experimentation (CONCEA). Mosquitoes were reared under controlled insectary conditions (27 ± 1 °C, 75–80% relative humidity, 12:12 h light:dark cycle), and only females aged 5–7 days post-emergence were used. Mosquito midguts were dissected under sterile conditions in phosphate-buffered saline (PBS, pH 7.4) using fine forceps under a stereomicroscope. Dissected tissues were homogenized in sterile PBS and plated on Brain Heart Infusion (BHI) agar, a nutrient-rich medium suitable for the growth of fastidious microorganisms. After incubation at 28 °C for 48 h, colonies displaying characteristic red pigmentation were selected and repeatedly streaked on fresh BHI agar to ensure clonal isolation. Pure colonies were preserved in frozen stocks at −70 °C.

Identification of *S. plymuthica* was performed by PCR amplification and sequencing of the 16S rRNA gene using universal primers (27F and 1492R). Sequences were analyzed using BLASTn against the NCBI database (accessed on 2 April 2025) and confirmed with ≥99.05% identity to *S. plymuthica* strains (accession number NR_114158.1).

*S. plymuthica* cells isolated from the midgut of *A. aegypti* were cultured in heme-free modified CASO medium (used as control and abbreviated as MC), which was prepared by modifying the standard CASO medium to exclude all heme sources. The medium composition was 15.0 g·L^−1^ casein peptone, 2.5 g·L^−1^ potassium phosphate, 5.0 g·L^−1^ sodium chloride, and 10.0 g·L^−1^ glucose. For solid media, agar was added at 15 g·L^−1^. Frozen stock cultures (−70 °C) were reactivated in 25 mL of MC broth and incubated at 28 °C for 12 h. A 5% (*v*/*v*) inoculum was then transferred to fresh MC broth (50 mL) and incubated at 28 °C, 110 rpm, until mid-log phase (OD_600_ = 0.6).

For solid medium assays, three sets of conditions were tested: (1) Hemin supplementation—Hemin (Sigma-Aldrich, St. Louis, MO, USA) was dissolved in 0.1 M NaOH and buffered with phosphate-buffered saline (PBS; 0.5 M NaH_2_PO_4_, 0.5 M Na_2_HPO_4_, 0.15 M NaCl, pH 7.4); (2) Inorganic iron supplementation—Ferric chloride and ferrous sulfate were dissolved in distilled water; (3) Blood derivatives—Defibrinated blood (4%) and hemoglobin (4 mg·mL^−1^, solubilized in sterile distilled water) were added to autoclaved medium when cooled to 55–60 °C.

All supplements were added to molten autoclaved MC medium to yield final concentrations of 5 µM, 10 µM, 0.5 mM, 1 mM, or 5 mM. For each condition, 20 mL of supplemented medium was poured into sterile Petri dishes (90 × 15 mm). Plates were spot-inoculated with 10 µL of mid-log phase *S. plymuthica* culture and incubated at 28 °C for 48 h. Colony diameter and pigmentation were quantified using ImageJ (NIH, v1.54d). Images of plates were acquired under standardized lighting conditions. For diameter measurement, images were converted to 8-bit, a scale was set using the plate diameter, and the “Analyze Particles” tool was used to measure colony area, which was converted to diameter (mm) using the formula: Diameter = 2 × √(Area/π). For pigmentation, the mean gray value was measured within a fixed region of interest (ROI) for each colony and normalized to the control. Three biological replicates (*n* = 3 plates per condition) with five colonies per plate were analyzed. Data are presented as mean ± SD. Statistical differences between conditions were assessed using one-way ANOVA with Tukey’s post-hoc test, with significance defined as *p* < 0.05.

For growth kinetics in liquid medium, colonies grown on MC plates were suspended in PGP broth (5.0 g·L^−1^ casein peptone, 2.0 g·L^−1^ potassium phosphate, 10 mL·L^−1^ glycerol, pH 7.0) and adjusted to OD_600_ = 0.1. Aliquots (2 µL) were inoculated into 96-well microplates containing 300 µL of MC broth supplemented with hemin at 5 µM, 10 µM, 0.5 mM, or 1 mM. OD_600_ readings were taken hourly over 17 h using a Bioscreen C™ plate reader (Thermolabsystems, Helsinki, Finland). Sterile medium was used as a blank control. Raw values were exported to Excel for growth curve generation. Statistical comparisons were performed using one-way ANOVA, with significance defined as *p* < 0.05.

### 2.2. Proteomic Analysis

#### 2.2.1. Obtaining of Bacterial Biomass

To mimic the high heme concentrations encountered in the mosquito midgut, *S. plymuthica* was cultured on solid medium supplemented with 5 mM hemin—a condition chosen to induce oxidative stress while supporting colony formation. Activated bacterial cultures were inoculated onto solid media containing 5 mM hemin. Five 10 µL aliquots were evenly distributed onto three Petri dishes and incubated at 28 °C for 48 h. Two colonies per plate were randomly selected, suspended in autoclaved ultrapure water, and centrifuged at 20,817 g for 4 min at 4 °C to remove residual hemin. This washing step was repeated three times. The bacterial cells were then frozen in liquid nitrogen and stored at −80 °C for 24 h before proteomic analysis.

#### 2.2.2. Protein Extraction

Total protein was extracted according to a previously described method [18], with modifications. Samples were treated with 1 mL of TCA/DTT/acetone buffer (10% TCA, 20 mM DTT in 100% acetone), stirred for 30 min at 4 °C, and incubated overnight at −20 °C. After centrifugation (23,000× *g*, 30 min, 4 °C), pellets were washed three times with cold acetone containing 20 mM DTT, vortexed, and centrifuged (12,000× *g*, 5 min, 4 °C). Dried pellets were suspended in 1 mL of solubilization buffer (7 M urea, 2 M thiourea, 1% DTT, 2% Triton X-100, 5 µM pepstatin, 1 mM PMSF), homogenized, and centrifuged (12,000× *g*, 15 min, 4 °C). Protein concentration was determined using the 2D-Quant Kit (GE Healthcare, Chicago, IL, USA).

#### 2.2.3. Protein Digestion

Protein digestion was performed as previously described [19]. A 100 μg protein aliquot was desalted using Amicon Ultra-0.5 3 kDa filters (Merck Millipore, Burlington, MA, USA) with 50 mM ammonium bicarbonate (pH 8.5) by centrifugation at 15,000× *g* for 10 min at 20 °C, repeated three times. The volume was reduced to ~50 μL. Proteins were denatured with 25 μL of 0.2% (*v*/*v*) RapiGest^®^ (Waters, Milford, MA, USA) at 80 °C for 15 min. Reduction was carried out with 2.5 μL of 100 mM DTT at 60 °C for 30 min, followed by alkylation with 2.5 μL of 300 mM iodoacetamide at room temperature (30 min, dark). Excess iodoacetamide was quenched with 5 μL of 100 mM DTT. Trypsin (20 μL, 50 ng/μL; Promega, Madison, WI, USA) was added, and digestion proceeded overnight at 37 °C. RapiGest^®^ was removed with 10 μL of 5% (*v*/*v*) TFA at 37 °C for 90 min, followed by centrifugation at 16,000× *g* for 30 min. The supernatant was transferred to glass tubes (Total Recovery, Waters).

#### 2.2.4. LC-MS/MS Analysis

Shotgun proteomics was performed using a nanoAcquity UPLC coupled to a Synapt G2-Si HDMS (Waters) for ESI-QTOF HDMSE analysis [19]. Digested samples (1 μg) were injected and normalized based on total ion counts from MSE scouting runs using ProteinLynx Global Server v3.0 (PLGS, Waters). Three biological replicates were analyzed. Chromatographic separation used a C18 trap column (180 μm × 20 mm, 5 μm; Waters) at 5 μL/min for 3 min, followed by an analytical HSS T3 column (100 μm × 100 mm, 1.8 μm; Waters) at 600 nL/min and 60 °C. The binary gradient was: 7% B (0–3 min), 7–40% B (3–93 min), 40–85% B (93–97 min), 85% B (97–101 min), 85–7% B (101–103 min), and 7% B until 120 min. Mobile phase A: 0.1% formic acid in water; B: 0.1% formic acid in acetonitrile. MS acquisition was in positive mode (35,000 FWHM, V mode) with ion mobility (wave velocity 600 m/s; helium and IMS gas at 180 and 90 mL/min). High-energy transfer collision energy ramped from 19–55 V. Cone and capillary voltages were 30 V and 2750 V; source temperature: 70 °C. TOF scans (0.5 s) were acquired in continuum mode (50–2000 Da). External calibration used 100 fmol/μL [Glu1]-fibrinopeptide B with lock mass every 30 s. Data were acquired using MassLynx v4.1.

#### 2.2.5. Proteomics Data Analysis

The mass spectrometry proteomics data have been deposited to the ProteomeXchange Consortium via the PRIDE [20] partner repository with the dataset identifier PXD062643. Raw data were processed with Progenesis QI for Proteomics v2.0 (Nonlinear Dynamics, Newcastle upon Tyne, UK). Database search parameters included one missed cleavage, minimum of two peptides per protein, fixed carbamidomethylation (C), variable oxidation (M), and phosphorylation (S, T, Y), 1% FDR (protein-level false discovery rate), score > 4 (Progenesis QI identification score threshold), and 10 ppm mass accuracy. Proteins were identified using the *S. plymuthica* S13 proteome (UniProtKB, 4987 entries). Only proteins detected in all three biological replicates were considered. Differentially accumulated proteins were determined via ANOVA (*p* < 0.05), with fold-change thresholds of >1.5 (upregulated) and <0.667 (downregulated).

Functional annotation used Blast2GO v3.0 PRO [21] and UniProtKB, while subcellular localization was predicted with FUEL-Mloc [22]. GO enrichment was performed using ShinyGO v0.82 [23] with the complete *S. plymuthica* proteome as background. Enriched GO terms were defined by FDR < 0.05 (Fisher’s exact test, Benjamini–Hochberg corrected) with ≥5 genes per term. Top 10 terms were visualized by fold enrichment, significance (−log10(FDR)), and gene count (bar size).

## 3. Results

### 3.1. Effects of Hemin Supplementation on S. plymuthica Growth

**Figure 1:**
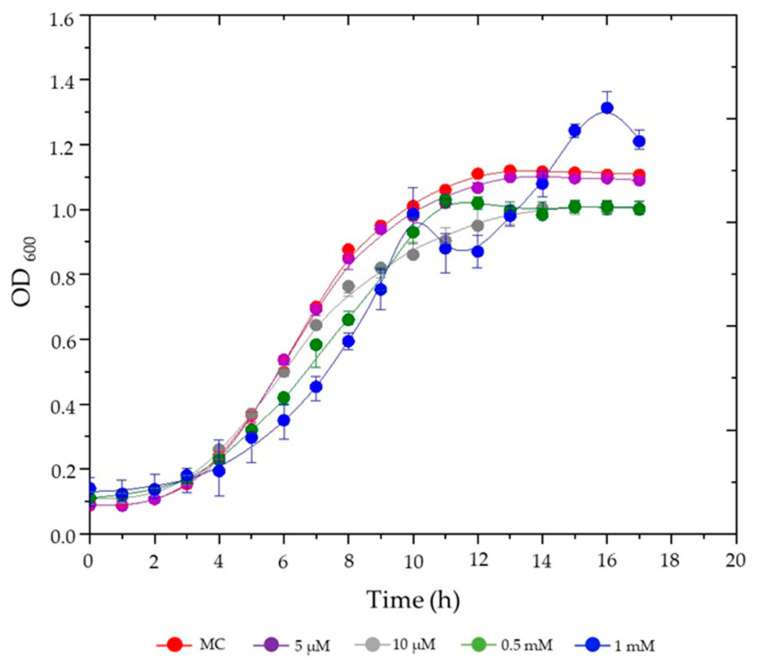
Growth kinetics of *S. plymuthica* under increasing hemin concentrations. *S. plymuthica* was cultured in MC (heme-free modified CASO medium (control; red) or in MC supplemented with 5 µM (purple), 10 µM (gray), 0.5 mM (green) or 1 mM (blue) hemin. Optical density at 600 nm (OD_600_) was recorded hourly for 17 h. Data points represent mean ± standard deviation (SD) of three independent cultures. Statistical significance refers to final OD_600_ comparisons at stationary phase (12–17 h). Error bars indicate SD; in some cases, error bars are smaller than the symbols.

The growth dynamics of *S. plymuthica* were evaluated in liquid culture media supplemented with various concentrations of hemin (5 µM, 10 µM, 0.5 mM, and 1 mM) compared to the heme-free control medium (MC) (Figure 1).

As shown in Figure 1, *S. plymuthica* exhibited a dose-dependent growth response to hemin. At the lowest concentration tested (5 μM), hemin had minimal impact on bacterial growth, with optical density profiles nearly identical to the control. Similarly, 10 μM hemin resulted in final OD_600_ values comparable to the control, though a slight reduction in growth rate was observed after 8 h. At the intermediate concentration (0.5 mM), *S. plymuthica* showed reduced growth rates throughout most of the cultivation period but achieved final biomass levels (OD_600_) similar to the control. In contrast, the highest hemin concentration (1.0 mM) induced the most pronounced effects: growth rates were suppressed during the first 10 h, followed by a distinct diauxic growth pattern characterized by two exponential phases separated by a lag period. This adaptation ultimately led to significantly enhanced biomass accumulation, with a maximum OD_600_ of ~1.3 compared to ~1.1 for the control (*p* < 0.05, ANOVA). The biphasic growth profile at 1.0 mM hemin suggests a metabolic adaptation process in which *S. plymuthica* initially experiences growth inhibition due to hemin-induced stress but subsequently exploits iron derived from hemin degradation to achieve superior biomass production. All other tested conditions followed standard growth phase transitions: a lag phase (~2–3 h), exponential growth (~3–11 h), and entry into stationary phase (~11–12 h).

**Figure 2:**
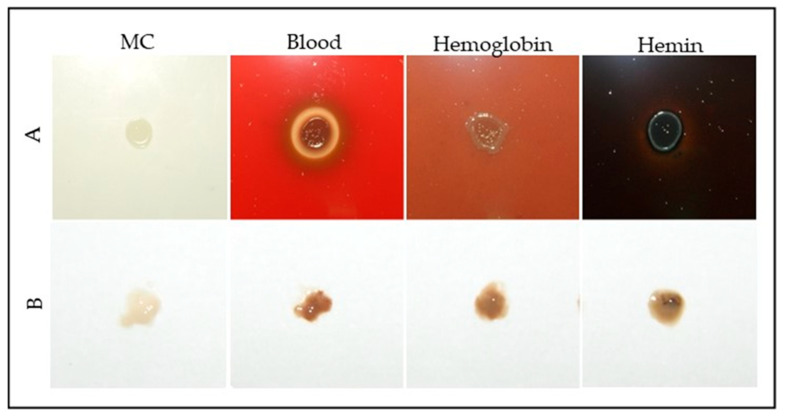
Growth of *S. plymuthica* on solid media supplemented with blood-derived heme sources. (**A**) Colonies of *S. plymuthica* grown for 48 h at 28 °C on MC (heme-free modified CASO solid medium; control), or MC supplemented with 4% defibrinated blood, 4 mg/mL hemoglobin, or 5 mM hemin. (**B**) Representative colony samples collected from each condition after incubation. Changes in pigmentation and morphology were visually assessed, with darker coloration observed in heme- and blood-supplemented media, consistent with heme accumulation or metabolism.

To further assess the effects of heme-related compounds, *S. plymuthica* was grown on solid heme-free modified CASO medium (MC) and MC supplemented with 4% blood, 4 mg/mL hemoglobin, or 5 mM hemin (Figure 2).

Colonies grown on blood, hemoglobin, or hemin-supplemented media displayed increased pigmentation and altered morphology compared to the control (Table S1; Supplementary Materials). Quantitative analysis using ImageJ revealed that the average colony diameter was 3.7 ± 0.3 mm for the control, 3.9 ± 0.2 mm for 4% blood, 3.7 ± 0.2 mm for 4 mg/mL hemoglobin, and 3.5 ± 0.3 mm for 5 mM hemin. The pigmentation index (normalized mean gray value) was 1.0 ± 0.1 for the control, 4.8 ± 0.4 for 4% blood, 5.2 ± 0.5 for hemoglobin, and 7.1 ± 0.6 for hemin. Colonies on hemoglobin and hemin media exhibited the most intense pigmentation, with hemin yielding the darkest coloration. Blood supplementation resulted in a brown halo around the colonies, suggestive of hemolytic activity or pigment diffusion. All heme-supplemented conditions showed significantly higher pigmentation compared to the control (one-way ANOVA with Tukey’s post-hoc test, *p* < 0.01), indicating that heme and hemoglobin are readily metabolized by *S. plymuthica*, supporting both growth and pigment production.

**Figure 3:**
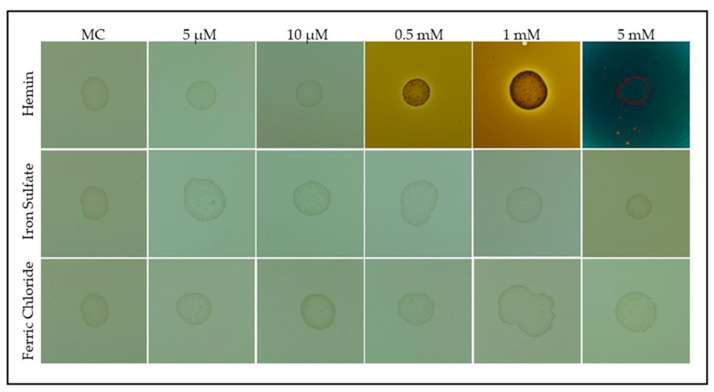
Growth of *S. plymuthica* on solid medium supplemented with heme and inorganic iron sources. *S. plymuthica* was grown for 48 h at 28 °C on MC (heme-free modified CASO solid medium; control) or MC supplemented with increasing concentrations of hemin (5 µM, 10 µM, 0.5 mM, 1 mM, and 5 mM), ferrous sulfate, or ferric chloride. Colony darkening intensified with higher hemin concentrations, indicating potential heme accumulation or active metabolism. No comparable phenotypic change was observed with inorganic iron salts, suggesting a specific response to heme.

To compare the effects of heme and inorganic iron sources, bacterial growth was evaluated on modified CASO medium supplemented with either hemin or iron salts (ferric chloride and iron sulfate) (Figure 3).

As shown in Figure 3, *S. plymuthica* formed colonies under all tested conditions. Quantitative analysis using ImageJ revealed that the average colony diameter (mean ± SD) was 4.2 ± 0.3 mm for the control, 4.1 ± 0.2 mm for 5 µM hemin, 4.0 ± 0.3 mm for 10 µM hemin, 4.5 ± 0.2 mm for 0.5 mM hemin, 4.7 ± 0.3 mm for 1 mM hemin, and 3.8 ± 0.4 mm for 5 mM hemin. For iron salts, colony diameters were 4.2 ± 0.3 mm (ferrous sulfate) and 4.3 ± 0.3 mm (ferric chloride), with a significant reduction observed at 5 mM ferrous sulfate compared to the control (one-way ANOVA with Tukey’s post-hoc test, *p* < 0.05) (Table S2, Supplementary Materials). Pigmentation index (normalized mean gray value, mean ± SD) was 1.0 ± 0.1 for the control, 1.1 ± 0.1 for 5 µM hemin, 1.2 ± 0.2 for 10 µM hemin, 3.8 ± 0.3 for 0.5 mM hemin, 5.9 ± 0.4 for 1 mM hemin, and 7.2 ± 0.5 for 5 mM hemin. Inorganic iron salts did not significantly alter pigmentation compared to the control (ferrous sulfate: 1.0 ± 0.1; ferric chloride: 1.0 ± 0.1; *p* > 0.05) (Table S2, Supplementary Materials). All concentrations of hemin ≥0.5 mM resulted in significantly increased pigmentation compared to the control (*p* < 0.01). These results indicate that while *S. plymuthica* can utilize both organic (hemin) and inorganic iron sources for growth, hemin is more efficiently assimilated and strongly stimulates pigmentation, supporting its role as a preferred iron source and metabolic regulator in this bacterium.

### 3.2. Effects of Hemin on Proteomic Profiles

Given the robust growth of *S. plymuthica* under high heme conditions, a proteomic analysis was conducted to elucidate molecular adaptations in response to 5 mM hemin. Cells were cultivated on solid medium to mimic the semi-solid environment of the mosquito midgut during blood digestion, where the blood bolus becomes concentrated as water is rapidly eliminated. A total of 436 proteins were identified, of which 82 (18.8%) were differentially accumulated—28 upregulated and 54 downregulated in the presence of hemin. Only proteins consistently detected across biological replicates were considered for downstream analyses. The complete mass spectrometry proteomics data have been deposited to the ProteomeXchange Consortium via the PRIDE partner repository with the dataset identifier PXD062643.

Subcellular localization predictions (FUEL-mLoc) indicated that upregulated proteins were predominantly cytoplasmic (42%) or periplasmic (28.6%), while downregulated proteins were primarily cytoplasmic (57.4%) or localized to the inner membrane (22%). Notably upregulated proteins included the universal stress protein (S4YPP8; 11.8-fold), flavodoxin (S4YFI2), and an ABC transporter ATPase (S4YNE7; 4.7-fold), which are associated with oxidative stress resistance and transport processes (Table 1).

Among the downregulated proteins (Table 1), 31 were found in the cytoplasm, including superoxide dismutase (SOD, S4YDE8), an antioxidant enzyme catalyzing the dismutation of superoxide radicals into oxygen and hydrogen peroxide. In the inner membrane, the multidrug transporter (S4YMN9), which facilitates cytotoxic compound export, was downregulated, suggesting altered membrane transport priorities. The extracellular porin protein (S4YLK3), involved in small molecule transport across the membrane, was also affected. Furthermore, the extracellular M15 peptidase (S4YLD0), responsible for protein hydrolysis, was downregulated. Two periplasmic proteins were identified among the downregulated proteins: a phenazine synthesis-related protein (S4YGX9), which may influence microbial competition and antibiotic production, and agmatinase (S4YS69), an enzyme involved in polyamine biosynthesis.

Among the downregulated proteins listed in Table 1, antioxidant enzymes such as superoxide dismutase (SOD; S4YDE8), and transporters such as the multidrug efflux protein (S4YMN9) and porin (S4YLK3) were reduced, indicating a shift in redox and membrane transport priorities. Enzymes involved in protein turnover and metabolic pathways (e.g., M15 peptidase S4YLD0, phenazine synthesis protein S4YGX9, and agmatinase S4YS69) were also downregulated, highlighting a broad suppression of cellular functions under heme stress.

**Figure 4:**
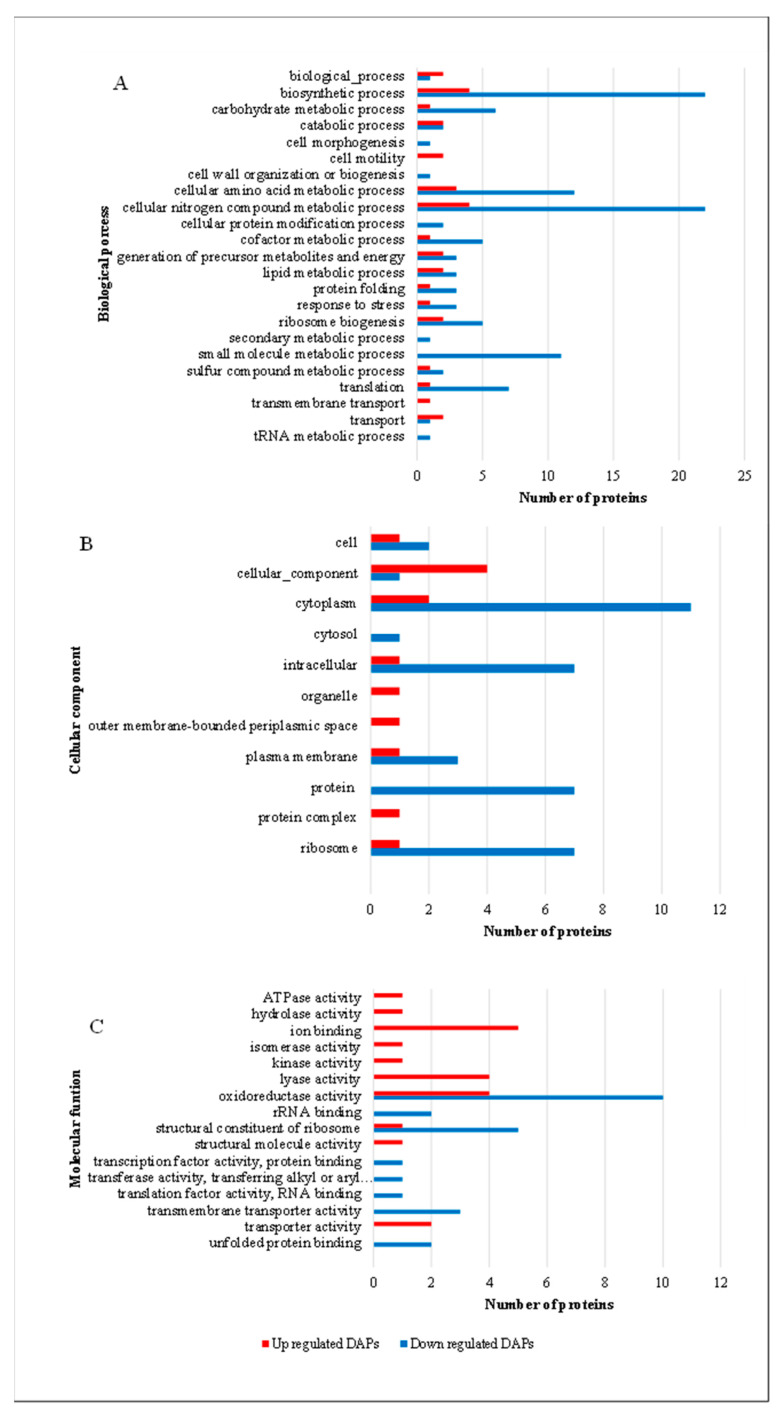
Functional classification of differentially abundant proteins (DAPs) in *S. plymuthica* under heme stress. Proteins differentially abundant in *S. plymuthica* cultured in the presence of heme were classified based on Gene Ontology (GO) annotations into (**A**) Biological Processes, (**B**) Cellular Components, and (**C**) Molecular Functions. The number of proteins associated with each category is represented by bar length. Red bars indicate upregulated proteins, while blue bars indicate downregulated proteins. Heme exposure was associated with prominent downregulation of proteins involved in translation, ribosome biogenesis, and redox functions, and the upregulation of proteins related to membrane transport and stress adaptation.

Gene ontology analysis using Blast2GO categorized differentially accumulated proteins into cellular components, biological processes, and molecular functions (Figure 4).

Upregulated proteins were associated with transmembrane transport—a process critical for heme acquisition and adaptation to oxidative stress. Downregulated proteins were linked to cellular morphogenesis, cell wall organization/biogenesis, tRNA metabolism, and secondary metabolism, reflecting reduced energy expenditure on non-essential functions. Upregulated proteins were enriched in the periplasmic space (~20-fold) and organelles, underscoring their role in heme transport and detoxification mechanisms. Molecular function analysis revealed that upregulated proteins exhibited lyase activity, ATPase activity, structural molecule activity, and ion binding, essential for stress adaptation and heme utilization. In contrast, downregulated proteins included translation factors and RNA-binding proteins, indicating reduced protein synthesis under stress conditions. These patterns were clearly reflected in the Gene Ontology (GO) enrichment profiles of differentially expressed proteins, as shown in Figure 4.

**Figure 5:**
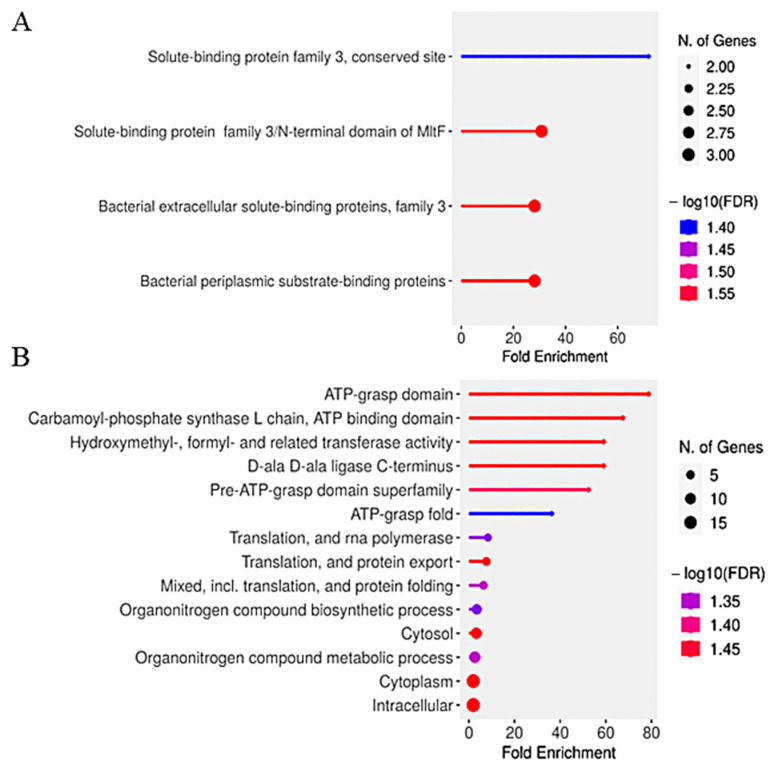
Functional enrichment analysis of differentially expressed genes in *S. plymuthica* exposed to heme. Bar plots show significantly enriched Gene Ontology (GO) terms (FDR < 0.05) among upregulated (**A**) and downregulated (**B**) genes in *S. plymuthica* following treatment with 5 mM hemin. The x-axis indicates fold enrichment, while bar height represents the number of genes annotated per term. Color intensity corresponds to statistical significance (−log_10_(FDR)).

Figure 5 illustrates the functional enrichment analysis of differentially expressed genes in *S. plymuthica* under heme stress.

Functional enrichment analysis of differentially expressed genes in *S. plymuthica* exposed to 5 mM hemin revealed distinct biological processes associated with upregulated and downregulated genes (Figure 5). Upregulated genes (Figure 5A) were significantly enriched for solute-binding and periplasmic transport categories, including “solute-binding protein family 3, conserved site” (~60-fold enrichment), “solute-binding protein family 3/N-terminal domain of MITF,” “bacterial extracellular solute-binding proteins, family 3,” and “bacterial periplasmic substrate-binding proteins.” These findings indicate a strong activation of heme acquisition and transport mechanisms, consistent with the upregulation of ABC transporters and substrate-binding proteins identified in our proteomic analysis (Table 1).

Downregulated genes (Figure 5B) showed significant enrichment for ATP-dependent processes, such as “ATP-grasp domain” (~70-fold enrichment), “carbamoyl-phosphate synthase L chain, ATP binding domain” (~60-fold), and “hydroxymethyl-, formyl- and related transferase activity” (~40-fold). Additional downregulated categories included “D-ala D-ala ligase C-terminus,” “translation and RNA polymerase,” and “organonitrogen compound biosynthetic/metabolic processes.” This pattern suggests suppression of energy metabolism, nitrogen compound biosynthesis, and protein synthesis under oxidative stress conditions.

Together, these results demonstrate that *S. plymuthica* adapts to high heme by upregulating genes involved in extracellular heme binding and transport, while downregulating energy-intensive and biosynthetic pathways. This metabolic reprogramming likely prioritizes heme detoxification and stress tolerance, supporting the bacterium’s survival in the oxidative environment of the mosquito midgut during blood digestion.

## 4. Discussion

During blood digestion, the midgut of *A. aegypti* is exposed to extreme oxidative conditions due to the release of free heme, which can reach concentrations of approximately 10 mM [8]. This level of heme is toxic to most bacteria, making survival in this environment a strong selective pressure.

In liquid growth media, *S. plymuthica* demonstrated remarkable tolerance to hemin concentrations up to 1 mM, with clear benefits to cell growth, indicating robust iron homeostasis and metabolic flexibility. This tolerance contrasts sharply with the sensitivity observed in model organisms such as *Escherichia coli* and *Staphylococcus aureus*, whose hemin tolerance thresholds are in the micromolar range [24,25]. The diauxic growth pattern observed at this concentration reflects a sophisticated metabolic adaptation to iron-rich environments. This biphasic response is consistent with well-documented bacterial strategies for coping with nutrient transitions and iron overload [26,27]. The initial rapid growth phase likely results from efficient hemin utilization as an iron source, while the subsequent lag phase reflects cellular adaptation to oxidative stress and iron excess, involving downregulation of iron uptake and activation of detoxification pathways [28]. After this adaptation, growth resumes with increased biomass, demonstrating the metabolic flexibility and unique strategies that enable *S. plymuthica* to thrive under high hemin concentrations.

The ability of *S. plymuthica* to grow robustly on solid media containing 5 mM hemin—the highest concentration tested and the maximum solubility limit under our experimental conditions—further underscores its exceptional tolerance. This concentration, which far exceeds the toxicity thresholds for most bacteria, was selected for our proteomic analysis to elucidate the molecular basis of this adaptation. The observed tolerance is not merely phenotypic but is supported by extensive proteomic remodeling, encompassing heme transport, ROS detoxification, and global metabolic reprogramming. These features underscore the ecological resilience of *S. plymuthica* in the mosquito midgut and highlight it as a model of oxidative stress adaptation. This resistance is likely mediated by the upregulation of ABC-type transporters, including S4YUX6 and S4YGS6, which may function in active heme import and/or efflux, as described for the HemTUV system in *Serratia marcescens* [29].

Additionally, the formation of halos around colonies grown on hemin-supplemented media, combined with the overexpression of hemophore-like proteins (e.g., S4YJK9), suggests that *S. plymuthica* employs an extracellular heme sequestration strategy to mitigate toxicity, likely by limiting intracellular accumulation. This approach contrasts with two well-characterized mechanisms in *Yersinia* species. In *Yersinia pestis*, heme is captured and stored in periplasmic aggregates via the hms locus, forming polymeric complexes that likely serve as iron reserves rather than immediate sources of nutrients [30,31]. Conversely, Y. enterocolitica utilizes periplasmic-binding transport (PBT) systems to import heme into the cytoplasm, where it is enzymatically degraded to release iron for metabolic use [32]. Compared to these models, the dynamic extracellular handling of heme observed in *S. plymuthica* appears to prioritize detoxification over iron acquisition, potentially offering an advantage in the highly oxidative, heme-rich environment of the mosquito midgut.

The coordinated upregulation of hemophore-like proteins and ABC transporters in *S. plymuthica* suggests a heme management strategy distinct from classical uptake systems. While structurally similar to the Has and Phu systems of *S. marcescens* [33,34] and *P. aeruginosa* [35], which mediate heme acquisition, the components in *S. plymuthica* may instead function to stabilize heme extracellularly, minimizing intracellular toxicity and oxidative stress. This adaptation could reflect a niche-specific response to heme overload in oxidative environments like the mosquito midgut.

Heme toxicity is intrinsically linked to ROS generation via Fenton chemistry [36]. To mitigate this, *S. plymuthica* appears to deploy alternative redox defense systems. The universal stress protein S4YPP8, strongly upregulated under heme stress, likely contributes to macromolecular protection during oxidative insult [37], while the induction of the flavodoxin S4YFI2 suggests a shift toward iron-sparing electron transfer, as flavodoxins can substitute for ferredoxins under oxidative or iron-limiting conditions [38,39]. Notably, *S. plymuthica* downregulates superoxide dismutase, diverging from other *Serratia* strains such as LCN16, which rely on SOD for ROS scavenging [15].

In addition to stress responses, *S. plymuthica* exhibits significant metabolic adjustments under heme-induced stress. Downregulation of ribosomal proteins and ATP synthase components indicates a strategic reduction in protein synthesis and oxidative phosphorylation, likely aimed at conserving energy and reducing endogenous ROS production [40]. Simultaneously, the upregulation of enzymes from the pentose phosphate pathway (PPP), including transketolase S4YN26, enhances NADPH production, a crucial cofactor for maintaining redox balance through antioxidant defenses [41]. This metabolic rerouting mirrors responses observed in *A. aegypti* midguts following blood feeding, where PPP activity sustains redox homeostasis [3].

The dominance of *S. plymuthica* during blood meal digestion suggests a mutualistic relationship with its mosquito host. By reducing heme toxicity and ROS accumulation, this symbiont may contribute to midgut integrity and epithelial protection, thereby enhancing host fitness [10,42]. Collectively, these findings reveal a multi-faceted and highly specialized response to heme stress, confirming the exceptional tolerance of *S. plymuthica* at both the physiological and molecular levels. Such robust adaptations are rare among gut-associated bacteria and position this symbiont as uniquely suited to thrive in the harsh, heme-rich midgut environment of hematophagous insects.

Altogether, our findings position *S. plymuthica* as a key symbiont in *A. aegypti*, equipped with sophisticated molecular tools to overcome the challenges of blood digestion. Its adaptive features not only support microbial persistence but may also shape mosquito biology and vector competence. These findings highlight the potential of *S. plymuthica* as a microbiota-based tool for mosquito control. By elucidating the molecular mechanisms underlying heme tolerance and oxidative stress adaptation, our study opens new perspectives for developing targeted interventions that manipulate the mosquito microbiota to reduce vector competence. Future research should explore the application of *S. plymuthica* in paratransgenesis or competitive exclusion strategies to disrupt pathogen transmission in mosquito populations.

## Figures and Tables

**Table 1:** Differentially abundant proteins (DAPs) in *S. plymuthica* under heme stress (5 mM), categorized by subcellular localization.

UniProt ID	LC	Protein Name	ANOVA	Fold Change
**Upregulated DAPs**				
S4YPP8	C	Universal stress protein	0.0003	11.8
S4YNE7	IM	ABC transporter	0.0008	4.7
S4YIR5	IM	Transcriptional regulator	0.0000	4.0
S4YJW6	EX	Flagellin homolog p5	0.0099	3.9
S4YUX6	IM	Amino acid ABC transporter substrate-binding (PAAT family)	0.0007	3.1
S4YLR1	C	Chorismate synthase	0.0099	2.3
S4YN26	C	Transketolase	0.0001	2.1
S4YPB0	P	Peptidylprolyl isomerase	0.0017	2.0
S4YKU6	C	Acetate kinase	0.0027	1.9
S4YQI6	C	Aspartate-semialdehyde dehydrogenase	0.0078	1.9
S4YF24	P	Hypothetical protein	0.0003	1.9
S4YQU9	C	Glycerol-3-phosphate dehydrogenase	0.0132	1.9
S4YKQ5	FL	Flagellar biosynthesis	0.0008	1.9
S4YGS6	P	ABC transporter substrate-binding	0.0005	1.8
S4YNH5	C	Keto-deoxy-phosphogluconate aldolase	0.0114	1.7
S4YJK9	P	ABC transporter substrate-binding	0.0020	1.7
S4YJR4	IM	Enhanced serine sensitivity	0.0018	1.7
S4YKW2	P	N-acetylglucosamine-6-phosphate deacetylase	0.0063	1.7
S4YIR6	C	Ribosome-binding factor A	0.0104	1.7
S4YQ21	P	D-ribose ABC transporter substrate-binding	0.0047	1.7
S4YPV1	C	O-succinylhomoserine (thiol)-lyase	0.0014	1.6
S4YAT2	C	Multifunctional fatty acid oxidation complex subunit alpha	0.0054	1.6
S4YDU9	C	Succinate dehydrogenase iron-sulfur subunit	0.0001	1.6
S4YTM2	IM	50S ribosomal protein L4	0.0038	1.6
S4YFI2	P	Flavodoxin	0.0133	1.6
S4YCZ0	IM	Lipo-related protein	0.0015	1.5
S4YGD8	P	Amino acid ABC transporter substrate-binding	0.0048	1.5
S4YJ39	C	Aconitate hydratase	0.0174	1.5
**Downregulated DAPs**				
S4YP79	P	50S ribosomal protein L30	0.0002	0.1
S4YDE8	C	Superoxide dismutase	0.0000	0.2
S4YJZ1	C	Keto-deoxy-phosphogluconate aldolase	0.0006	0.2
S4YN10	IM	F0F1 ATP synthase subunit delta	0.0020	0.2
S4YMN3	P	Glycine betaine ABC transporter substrate-binding	0.0265	0.3
S4YTP0	OM	cAMP regulatory protein	0.0000	0.3
S4YMN9	IM	Multidrug export protein	0.0008	0.4
S4YEZ3	IM	Cyclic di-GMP-binding family protein	0.0000	0.4
S4YNM7	C	Family transcriptional regulator	0.0002	0.4
S4YPD4	IM	30S ribosomal protein S4	0.0032	0.4
S4YLK3	OM	Porin, Gram-negative type	0.0000	0.4
S4YRN2	C	Sigma-24 factor	0.0010	0.4
S4YS69	P	Agmatinase	0.0002	0.4
S4YLB8	P	Ribosome hibernation promoting factor Hpf	0.0012	0.4
S4YJS5	P	30S ribosomal protein S16	0.0071	0.4
S4YM12	C	Cytosine deaminase	0.0004	0.4
S4YCV5	C	Oxidoreductase	0.0008	0.4
S4YXW7	IM	Glutamate racemase	0.0195	0.4
S4YB57	C	Malate dehydrogenase	0.0005	0.5
S4YNW0	C	Acetyl-coA carboxylase biotin carboxylase subunit	0.0449	0.5
S4YJI5	C	Phosphoribosylglycinamide formyltransferase 2	0.0002	0.5
S4YLD0	EX	Peptidase M15	0.0348	0.5
S4YD16	C	Adenylate kinase	0.0011	0.5
S4YL10	C	Aldehyde reductase	0.0012	0.5
S4YK92	OM	Transcriptional regulator	0.0165	0.5
S4YE80	C	GMP reductase	0.0332	0.5
S4YFE3	C	Leucine–tRNA ligase	0.0010	0.5
S4YJJ1	C	L-serine dehydratase 1	0.0017	0.5
S4YK46	C	Ribosome recycling factor	0.0011	0.5
S4YAD8	C	Mannitol-1-phosphate 5-dehydrogenase	0.0013	0.5
S4YD37	IM	Phosphatidylserine decarboxylase	0.0204	0.6
S4YE23	C	Delta-aminolevulinic acid dehydratase	0.0002	0.6
S4YHN7	C	Bifunctional UDP-glucuronic acid oxidase/UDP-4-amino-4-deoxy-L-arabinose formyltransferase	0.0058	0.6
S4YG49	C	Molecular chaperone	0.0039	0.6
S4YNM9	C	Elongation factor G	0.0026	0.6
S4YMR5	C	Ferredoxin–NADP(+) reductase	0.0401	0.6
S4YAU6	IM	50S ribosomal protein L10	0.0000	0.6
S4YDZ9	P	Carbamoyl phosphate synthase large subunit	0.0002	0.6
S4YN87	C	Pyruvate dehydrogenase (acetyl-transferring) homodimeric type	0.0052	0.6
S4YD04	P	Phosphoribosylamine–glycine ligase	0.0002	0.6
S4YIF2	P	50S ribosomal protein L7/L12	0.0015	0.6
S4YFK9	IM	Succinate dehydrogenase flavoprotein subunit	0.0000	0.6
S4YDV0	C	Glucose-1-phosphate thymidylyltransferase	0.0404	0.6
S4YC66	C	Urocanate hydratase	0.0003	0.6
S4YN76	C	Glutathione S-transferase	0.0001	0.6
S4YJV4	P	Serine/threonine kinase	0.0295	0.6
S4YH64	C	Transcriptional regulator	0.0302	0.7
S4YJJ2	C	Acetolactate synthase isozyme 3 small subunit	0.0168	0.7
S4YF25	C	Threonine synthase	0.0000	0.7
S4YG26	C	Peptidylprolyl isomerase	0.0003	0.7
S4YGX9	F	Phenazine biosynthesis family protein	0.0076	0.7
S4YV94	IM	Hypothetical protein	0.0216	0.7
S4YMD1	C	Universal stress protein	0.0073	0.7
S4YDU1	C	60 kDa chaperonin	0.0015	0.7

DAPs identified in *S. plymuthica* cells cultured in the presence of heme. Subcellular localization by prediction analysis using FUEL-mLoc. LC: Predicted cellular localization of the protein, IM: inner membrane, P: periplasm, OM: outer membrane; C: cytoplasm; EX: extracellular; FL: flagellum; F: fimbria.

## Data Availability

The mass spectrometry proteomics data have been deposited to the ProteomeXchange Consortium via the PRIDE19 partner repository with the dataset identifier PXD062643.

## Supplementary Materials

The following supporting information can be downloaded at: https://www.mdpi.com/article/10.3390/life15060950/s1, Table S1: Quantitative analysis of *S. plymuthica* colony diameter and pigmentation on solid media supplemented with blood-derived heme sources; Table S2: Quantitative analysis of *S. plymuthica* colony diameter and pigmentation on solid media supplemented with hemin and inorganic iron.

## References

[1] Gómez M., Martinez D., Muñoz M., Ramírez J.D. (2022). *Aedes aegypti* and *Aedes albopictus* microbiome/virome: New strategies for controlling arboviral transmission?. Parasit. Vectors.

[2] Talyuli O.A.C., Bottino-Rojas V., Polycarpo C.R., Oliveira P.L., Paiva-Silva G.O. (2021). Non-immune traits triggered by blood intake impact vectorial competence. Front. Physiol..

[3] Oliveira J.H., Gonçalves R.L., Lara F.A., Dias F.A., Gandara A.C., Menna-Barreto R.F., Edwards M.C., Laurindo F.R., Silva-Neto M.A., Sorgine M.H. (2011). Blood meal-derived heme decreases ROS levels in the midgut of *Aedes aegypti* and allows proliferation of intestinal microbiota. PLoS Pathog..

[4] Oliveira J.H.M., Talyuli O.A.C., Goncalves R.L.S., Paiva-Silva G.O., Sorgine M.H.F., Alvarenga P.H., Oliveira P.L. (2017). Catalase protects *Aedes aegypti* from oxidative stress and increases midgut infection prevalence of Dengue but not Zika. PLoS Negl. Trop. Dis..

[5] Orozimbo K.B.D.S., Tauil D.D.S.G., Licurgo A.M., Moreira F.F., Araújo J.D.S., Bertonceli M.A.A., Seabra S.H., Machado O.L.T., Lemos F.J.A. (2025). Structural and functional analysis of hemoglobin binding to the peritrophic matrix during blood digestion in *Aedes aegypti*. Insects.

[6] Bottino-Rojas V., Talyuli O.A., Jupatanakul N., Sim S., Dimopoulos G., Venancio T.M., Bahia A.C., Sorgine M.H., Oliveira P.L., Paiva-Silva G.O. (2015). Heme signaling impacts global gene expression, immunity and dengue virus infectivity in *Aedes aegypti*. PLoS ONE.

[7] Pascoa V., Oliveira P.L., Dansa-Petretski M., Silva J.R., Alvarenga P.H., Jacobs-Lorena M., Lemos F.J. (2002). *Aedes aegypti* peritrophic matrix and its interaction with heme during blood digestion. Insect Biochem. Mol. Biol..

[8] Eggleston H., Adelman Z.N. (2020). Transcriptomic analyses of *Aedes aegypti* cultured cells and ex vivo midguts in response to an excess or deficiency of heme: A quest for transcriptionally-regulated heme transporters. BMC Genom..

[9] Liu H., Yin J., Huang X., Zang C., Zhang Y., Cao J., Gong M. (2024). Mosquito gut microbiota: A review. Pathogens.

[10] Gaio A.O., Gusmão D.S., Santos A.V., Berbert-Molina M.A., Pimenta P.F., Lemos F.J. (2011). Contribution of midgut bacteria to blood digestion and egg production in *Aedes aegypti* (Diptera: Culicidae) (L.). Parasit. Vectors.

[11] Gusmão D.S., Santos A.V., Marini D.C., Bacci M., Berbert-Molina M.A., Lemos F.J. (2010). Culture-dependent and culture-independent characterization of microorganisms associated with *Aedes aegypti* (Diptera: Culicidae) (L.) and dynamics of bacterial colonization in the midgut. Acta Trop..

[12] Kulkova I., Wróbel B., Dobrzyński J. (2024). *Serratia* spp. as plant growth-promoting bacteria alleviating salinity, drought, and nutrient imbalance stresses. Front. Microbiol..

[13] Nordstedt N.P., Jones M.L. (2021). Genomic analysis of *Serratia plymuthica* MBSA-MJ1: A plant growth promoting rhizobacteria that improves water stress tolerance in greenhouse ornamentals. Front. Microbiol..

[14] Kakani P., Gupta L., Kumar S. (2020). Heme-Peroxidase 2, a peroxinectin-like gene, regulates bacterial homeostasis in *Anopheles stephensi* midgut. Front. Physiol..

[15] Vicente C.S., Nascimento F.X., Ikuyo Y., Cock P.J., Mota M., Hasegawa K. (2016). The genome and genetics of a high oxidative stress tolerant *Serratia* sp. LCN16 isolated from the plant parasitic nematode *Bursaphelenchus xylophilus*. BMC Genom..

[16] Wang H., Liu H., Peng H., Wang Y., Zhang C., Guo X., Wang H., Liu L., Lv W., Cheng P. (2022). A symbiotic gut bacterium enhances *Aedes albopictus* resistance to insecticide. PLoS Negl. Trop. Dis..

[17] Ramirez J.L., Souza-Neto J., Torres Cosme R., Rovira J., Ortiz A., Pascale J.M., Dimopoulos G. (2012). Reciprocal tripartite interactions between the *Aedes aegypti* midgut microbiota, innate immune system and dengue virus influences vector competence. PLoS Negl. Trop. Dis..

[18] Damerval C., De Vienne D., Zivy M., Thiellement H. (1986). Technical improvements in two-dimensional electrophoresis increase the level of genetic variation detected in wheat-seedling proteins. Electrophoresis.

[19] Reis R.S., Vale E.M., Heringer A.S., Santa-Catarina C., Silveira V. (2016). Putrescine induces somatic embryo development and proteomic changes in embryogenic callus of sugarcane. J. Proteom..

[20] Perez-Riverol Y., Bandla C., Kundu D.J., Kamatchinathan S., Bai J., Hewapathirana S., John N.S., Prakash A., Walzer M., Wang S. (2025). The PRIDE database at 20 years: 2025 update. Nucleic Acids Res..

[21] Conesa A., Götz S., García-Gómez J.M., Terol J., Talón M., Robles M. (2005). Blast2GO: A universal tool for annotation, visualization and analysis in functional genomics research. Bioinformatics.

[22] Wan S., Mak M.W., Kung S.Y. (2017). FUEL-mLoc: Feature-unified prediction and explanation of multi-localization of cellular proteins in multiple organisms. Bioinformatics.

[23] Ge S.X., Jung D., Yao R. (2020). ShinyGO: A graphical enrichment tool for animals and plants. Bioinformatics.

[24] Fox D., Asadollahi K., Samuels I., Spicer B., Kropp A., Lupton C., Lim K., Wang C., Venugopal H., Dramicanin M. (2024). Inhibiting heme-piracy by pathogenic Escherichia coli using de novo-designed proteins. bioRxiv.

[25] Nitzan Y., Ladan H., Malik Z. (1987). Growth-inhibitory effect of hemin on staphylococci. Curr. Microbiol..

[26] Akhter F., Womack E., Vidal J.E., Le Breton Y., McIver K.S., Pawar S., Eichenbaum Z. (2020). Hemoglobin stimulates vigorous growth of Streptococcus pneumoniae and shapes the pathogen’s global transcriptome. Sci. Rep..

[27] Lan C.Q., Oddone G., Mills D.A., Block D.E. (2006). Kinetics of Lactococcus lactis growth and metabolite formation under aerobic and anaerobic conditions in the presence or absence of hemin. Biotechnol. Bioeng..

[28] Torres V.J., Stauff D.L., Pishchany G., Bezbradica J.S., Gordy L.E., Iturregui J., Anderson K.L., Dunman P.M., Joyce S., Skaar E.P. (2007). A Staphylococcus aureus regulatory system that responds to host heme and modulates virulence. Cell Host Microbe..

[29] Létoffé S., Delepelaire P., Wandersman C. (2008). Functional differences between heme permeases: *Serratia marcescens* HemTUV permease exhibits a narrower substrate specificity (restricted to heme) than the Escherichia coli DppABCDF peptide-heme permease. J. Bacteriol..

[30] Perry R.D., Pendrak M.L., Schuetze P. (1990). Identification and cloning of a hemin storage locus involved in the pigmentation phenotype of *Yersinia pestis*. J. Bacteriol..

[31] Perry R.D., Lucier T.S., Sikkema D.J., Brubaker R.R. (1993). Storage reservoirs of hemin and inorganic iron in *Yersinia pestis*. Infect. Immun..

[32] Stojiljkovic I., Hantke K. (1994). Transport of haemin across the cytoplasmic membrane through a haemin-specific periplasmic binding-protein-dependent transport system in *Yersinia enterocolitica*. Mol. Microbiol..

[33] Cwerman H., Wandersman C., Biville F. (2006). Heme and a five-amino-acid hemophore region form the bipartite stimulus triggering the has signaling cascade. J. Bacteriol..

[34] Benevides-Matos N., Biville F. (2010). The Hem and Has haem uptake systems in *Serratia marcescens*. Microbiology.

[35] Dent A.T., Wilks A. (2020). Contributions of the heme coordinating ligands of the *Pseudomonas aeruginosa* outer membrane receptor HasR to extracellular heme sensing and transport. J. Biol. Chem..

[36] Winterbourn C.C. (1995). Toxicity of iron and hydrogen peroxide: The Fenton reaction. Toxicol. Lett..

[37] Luo D., Wu Z., Bai Q., Zhang Y., Huang M., Huang Y., Li X. (2023). Universal Stress Proteins: From Gene to Function. Int. J. Mol. Sci..

[38] Erdner D.L., Anderson D.M. (1999). Ferredoxin and flavodoxin as biochemical indicators of iron limitation during open-ocean iron enrichment. Limnol. Oceanogr..

[39] Tognetti V.B., Zurbriggen M.D., Morandi E.N., Fillat M.F., Valle E.M., Hajirezaei M.R., Carrillo N. (2007). Enhanced plant tolerance to iron starvation by functional substitution of chloroplast ferredoxin with a bacterial flavodoxin. Proc. Natl. Acad. Sci. USA.

[40] Imlay J.A. (2003). Pathways of oxidative damage. Annu. Rev. Microbiol..

[41] Kruger N.J., von Schaewen A. (2003). The oxidative pentose phosphate pathway: Structure and organisation. Curr. Opin. Plant Biol..

[42] Martinson V.G., Strand M.R. (2021). Diet–Microbiota Interactions Alter Mosquito Development. Front. Microbiol..

