# Supplementary material for: Exceptional Heme Tolerance in Serratia plymuthica: Proteomic Insights into Oxidative Stress Adaptation in the Aedes aegypti Midgut
Source: Life (Basel). 2025 Jun 13;15(6):950. doi: 10.3390/life15060950 (PMC12193951; doi:10.3390/life15060950)
Supplement: Supplementary file 1 [file life-15-00950-s001.zip › life-3636056-supplementary.pdf]

life

MDPI

Supplementary Materials

Table S1. Quantitative analysis of *S. plymuthica* colony diameter and pigmentation on solid media supplemented with blood-derived heme sources

Condition	Colony Diameter (mm)	Pigmentation Index
Control (MC)	3.7 ± 0.3	1.0 ± 0.1
4% Blood	3.9 ± 0.2*	4.8 ± 0.4*
4 mg/mL Hemoglobin	3.7 ± 0.2	5.2 ± 0.5*
5 mM Hemin	3.5 ± 0.3*	7.1 ± 0.6*

Colony diameter and pigmentation index of *S. plymuthica* grown for 48 h at 28 °C on heme-free modified CASO medium (MC, Control), or MC medium supplemented with 4% defibrinated blood, 4 mg/mL hemoglobin, or 5 mM hemin. Colony diameter (mm) was measured using ImageJ, and pigmentation index was calculated as the normalized mean gray value relative to the control. Data are presented as mean ± SD (n = 3). Asterisks indicate significant difference from control (ANOVA, $p < 0.01$).

Table S2. Quantitative analysis of *S. plymuthica* colony diameter and pigmentation on solid media with hemin and inorganic iron.

Condition	Colony Diameter (mm)	Pigmentation Index
Control	4.2 ± 0.3	1.0 ± 0.1
5 μM Hemin	4.1 ± 0.2	1.1 ± 0.1
10 μM Hemin	4.0 ± 0.3	1.2 ± 0.2
0.5 mM Hemin	4.5 ± 0.2	3.8 ± 0.3*
1 mM Hemin	4.7 ± 0.3	5.9 ± 0.4*
5 mM Hemin	3.8 ± 0.4	7.2 ± 0.5*
Ferrous Sulfate	4.2 ± 0.3	1.0 ± 0.1
Ferric Chloride	4.3 ± 0.3	1.0 ± 0.1

Colony diameter and pigmentation index were measured using ImageJ. Pigmentation index is normalized to control (1.0). Data are mean ± SD (n = 3). Asterisks indicate significant difference from control (ANOVA, $p < 0.01$).
